# Unusual Placement of an EBV Epitope into the Groove of the Ankylosing Spondylitis-Associated HLA-B27 Allele Allows CD8+ T Cell Activation

**DOI:** 10.3390/cells8060572

**Published:** 2019-06-11

**Authors:** Valentina Tedeschi, Josephine Alba, Fabiana Paladini, Marino Paroli, Alberto Cauli, Alessandro Mathieu, Rosa Sorrentino, Marco D’Abramo, Maria Teresa Fiorillo

**Affiliations:** 1 Department of Biology and Biotechnology ‘Charles Darwin’, Sapienza University of Rome, 00185 Rome, Italy; valentina.tedeschi@uniroma1.it (V.T.); fabiana.paladini@uniroma1.it (F.P.); rosa.sorrentino@uniroma1.it (R.S.); 2 Department of Chemistry, Sapienza University of Rome, 00185 Rome, Italy; josephine.alba@uniroma1.it; 3 Division of Clinical Immunology and Rheumatology, Department of Biotechnology and Medical Surgical Sciences, Sapienza University of Rome, 00185 Rome, Italy; marino.paroli@uniroma1.it; 4 Rheumatology Unit, Department of Medical Sciences and Public Health, University and AOU of Cagliari, Monserrato, 09042 Cagliari, Italy; cauli@unica.it (A.C.); amath@unica.it (A.M.)

**Keywords:** HLA-B27, viral peptides, computational analysis, ankylosing spondylitis

## Abstract

The human leukocyte antigen HLA-B27 is a strong risk factor for Ankylosing Spondylitis (AS), an immune-mediated disorder affecting axial skeleton and sacroiliac joints. Additionally, evidence exists sustaining a strong protective role for HLA-B27 in viral infections. These two aspects could stem from common molecular mechanisms. Recently, we have found that the HLA-B*2705 presents an EBV epitope (pEBNA3A-RPPIFIRRL), lacking the canonical B27 binding motif but known as immunodominant in the HLA-B7 context of presentation. Notably, 69% of B*2705 carriers, mostly patients with AS, possess B*2705-restricted, pEBNA3A-specific CD8+ T cells. Contrarily, the non-AS-associated B*2709 allele, distinguished from the B*2705 by the single His116Asp polymorphism, is unable to display this peptide and, accordingly, B*2709 healthy subjects do not unleash specific T cell responses. Herein, we investigated whether the reactivity towards pEBNA3A could be a side effect of the recognition of the natural longer peptide (pKEBNA3A) having the classical B27 consensus (KRPPIFIRRL). The stimulation of PBMC from B*2705 positive patients with AS in parallel with both pEBNA3A and pKEBNA3A did not allow to reach an unambiguous conclusion since the differences in the magnitude of the response measured as percentage of IFNγ-producing CD8+ T cells were not statistically significant. Interestingly, computational analysis suggested a structural shift of pEBNA3A as well as of pKEBNA3A into the B27 grooves, leaving the A pocket partially unfilled. To our knowledge this is the first report of a viral peptide: HLA-B27 complex recognized by TCRs in spite of a partially empty groove. This implies a rethinking of the actual B27 immunopeptidome crucial for viral immune-surveillance and autoimmunity.

## 1. Introduction 

The antigen processing and presentation pathway enables MHC class I molecules, in humans named Human Leukocyte antigens (HLAs), to alert CD8+ T lymphocytes as part of the immune-surveillance program [1]. To this aim, HLA class I molecules, expressed on the surface of almost all cells, display short peptides, usually nine residues in length, originating from endogenously synthesized microbial or cellular proteins, which are recognized by CD8+ T cells through their T cell receptors (TCRs) [2,3].

These peptide fragments are generated by proteasome degradation in the cytosol and the portion able to escape the shredding by cytosolic aminopeptidases, is translocated into the endoplasmic reticulum (ER) through the transporter associated with antigen processing (TAP) proteins [2]. Inside the ER, two resident aminopeptidases, ERAP1 and ERAP2, further shorten the peptides at the N-terminally extended end prior to loading into the HLA class I alleles [4,5].

The HLA class I molecules are heterotrimeric proteins of a polymorphic heavy chain assembling with the invariant beta2-microglobulin whose stability is strongly dependent on the presence of a bound peptide allowing the complex to reach a closed conformation. Hence, the bound peptide sits into a groove made by (A–F) pockets of the heavy chain where a network of interactions via hydrogen bonds and salt bridges ensures that the N- and C-termini and the main chain of the peptide fragment are anchored [6,7]. There is considerable variability in the contacts between the side chains of the peptide residues and the polymorphic pockets among all different HLA class I molecules whereas the N- and C-terminal interactions are well conserved [7,8]. Accordingly, thermodynamic data suggest that these interactions could be crucial for the formation and stabilization of the peptide/HLA class I complexes [9].

Among HLA class I alleles, the HLA-B27 is the focus of intense investigation given its involvement in some autoimmune disorders such as Ankylosing Spondylitis, the prototype of a group of chronic rheumatic inflammatory diseases named Spondyloarthropathies and, in the immune protection against some viral infections such as HIV, HCV, EBV, and Influenza virus [10,11,12,13]. Nevertheless, the molecular mechanisms linking the HLA-B27 to autoimmunity and to antiviral defence are still largely undefined. Such a duality could stem from overlapping pathways related directly or indirectly to the quality of its peptide repertoire [14]. Further evidence in this direction comes from genome wide association studies (GWAS) pointing out that both Endoplasmic Reticulum aminopeptidase 1 (ERAP1), which is in an epistatic relationship with the HLA-B27, and ERAP2, are risk factors for Ankylosing Spondylitis (AS) [15,16,17]. It is well acknowledged that both ER resident enzymes have a central role in shaping the immunopeptidome of HLA-B27 molecules [4,5]. Notably, ERAP1 allelic variants conferring a higher enzymatic activity as well as the presence of ERAP2 have been shown to be predisposing factors for AS [4,14,16,17].

The HLA-B27 immunopeptidome is characterized by the preferred binding of 9-mer peptides with an arginine at second position (P2) and hydrophobic or basic residues at their C-termini [13,18,19]. However, our and other groups have shown an expansion of the B27 repertoire to peptides with glutamine or lysine at the primary anchor position at P2 [20,21]. Moreover, several studies have highlighted the high flexibility of the HLA-B27 groove which can even accommodate longer peptides, either N- or C-terminally extended, whose availability is expected to be influenced by the allelic specificity and presence/absence of ERAP enzymes [22,23].

At present, more than 170 different HLA-B27 subtypes have been identified. The HLA-B*2705 is the ancestral allele which shows a worldwide association with AS [24]. Interestingly, some other HLA-B27 alleles, such as HLA-B*2709, relatively frequent in Sardinia (20% of all B27 alleles), do not predispose to the disease [25,26,27]. It is relevant that the HLA-B*2705 and B*2709 alleles differ by the unique polymorphism at aa 116 (Asp to His) which is positioned in the F pocket of the peptide binding groove [28]. This single substitution is critical for the peptide consensus sequence motifs, for the structural and dynamic features as well as for the T cell repertoire distinguishing the two B27 alleles [28,29,30].

Very recently, we have described in a high number of HLA-B*2705 subjects, mostly patients with AS, the presence of a HLA-B27-restricted CD8+ T cell reactivity against an EBV epitope from EBNA3A (pEBNA3A; RPPIFIRRL) which does not have a proper B27 binding consensus motif. Notably, the B*2709 allele is not a presenting molecule for such a peptide and consequently, in B*2709 positive individuals this immune response does not occur [31].

Herein, we investigate whether the reactivity towards pEBNA3A in the HLA-B*2705 context of presentation is a consequence of the recognition of the longer peptide KRPPIFIRRL (pKEBNA3A) endowed with a canonical B27 binding motif. Moreover, by computational approaches, we model the structural and dynamic behaviour of these complexes and discuss how the pEBNA3A and pKEBNA3A peptides fit into the groove of the B*2705 and the B*2709 alleles.

## 2. Materials and Methods

### 2.1. Study Subjects

Eighty-five HLA-B*2705-positive subjects (74 AS-patients and 11 HD) and nine HLA-B*2709-positive controls were enrolled in this study. Diagnosis of AS has been made according to modified New York criteria. The HLA-B*27 subtype was determined by serological analysis using ME1 mAb (specificity: HLA-B*27; -B*07; -B*42; -B*67; -B*73 and -B*w22) and genomic analysis. The study received the approval by the Ethics Committee of the University of Cagliari (365/09/CE). All subjects provided written informed consent prior to enrolment.

### 2.2. Synthetic Peptides

pEBNA3A (RPPIFIRRL; aa 379−387), an immunodominant EBV epitope restricted by HLA-B*07 [32] and its N-terminal-extended version pKEBNA3A (KRPPIFIRRL) have been used in this study. For the binding assay, a self-peptide restricted by HLA-B*27 (TIS, RRLPIFSRL; aa 325−333) [31] and an immunodominant EBV-epitope HLA-B*35-restricted (YPLHEQHGM; aa 458−466) were included. Peptides (purity > 95%) were purchased by Aurogene (Rome, Italy).

### 2.3. Cell Lines

TAP-defective CEM 174.T2 cells (ATCC number: CRL-1992™) and HMy2.C1R cells (ATCC number: CRL-1993™), stably expressing B*2705 or B*2709 were used. Cells were cultured in heat-inactivated 10% fetal bovine serum (FBS; Euroclone Spa, Pero, Milan, Italy)/RPMI 1640 medium (Euroclone Spa, Pero, Milan, Italy) supplemented with 2 mmol/L L-glutamine, 100 U/mL penicillin, 100 μg/mL streptomycin. T2 and C1R stable transfectants were maintained in medium added with G418 (800 μg/mL) or hygromycin B (200 μg/mL), respectively.

### 2.4. PBMC Stimulation

PBMC (4 × 10^6^) separated from whole blood by density gradient separation (Cedarlane Laboratories Ltd., Burlington, ON, Canada) were incubated with the indicated peptides (20 μM) and cultured for 12 days at 2 × 10^6^ cells/mL in 5% FBS/RPMI complete medium, and 10 U/mL of human recombinant interleukin 2 (IL2) (Roche Applied Science, Penzberg, Germany). On day 3 and 9, fresh medium containing 20 U/mL of IL2 was replenished. After 12 days of antigen pulsing, PBMC were used for intracellular IFNγ staining.

### 2.5. Intracellular IFNγ Staining

Briefly, C1R transfectants were incubated overnight with pEBNA3A or pKEBNA3A (30 μM) or with medium alone and thoroughly washed before to be plated with antigen-stimulated PBMC at 5:1 PBMC/APC ratio. After 1 h, the cells were treated with brefeldin A (10 μg/mL) at 37 °C for 16 h. Cells were stained with the anti-CD8-FITC mAb (BioLegend, San Diego, CA, USA), 20′ on ice, fixed with 4% paraformaldehyde 20′ on ice, permeabilized with 1% BSA/0.1% saponin in PBS 1X for 5′ at RT and then resuspended in 2% paraformaldehyde/1% BSA. Finally, cells were stained by an anti-IFNγ-APC mAb (BioLegend, San Diego, CA, USA) for 20′ at RT. The samples were acquired by a FACSCalibur flow cytometer (Becton Dickinson, Franklin Lakes, NJ, USA) and analysed by FlowJo software (Tree Star Inc, Ashland, OR, USA).

### 2.6. T2.B27 Stabilization Assay

The capability of the selected peptides to stabilize the B*2705 and B*2709 molecules on the cell surface of T2 stable transfectants was assessed according to the procedure previously described by Tedeschi et al. [31]. The surface amount of B27 molecules on T2 transfectants was determined by using ME1 mAb and a rabbit anti-mouse IgG-FITC (Jackson ImmunoResearch Europe, Suffolk, UK) as secondary antibody. An antibody of IgG isotype was also employed as control. The results are shown as the mean ± SD of three independent experiments.

### 2.7. Statistics

Data obtained by IFNγ-production assays from pEBNA3A- or pKEBNA3A-stimulated PBMCs from B*2705-positive carriers were compared by Mann Whitney U-test; the pEBNA3A response in B*2705- or B*2709-positive subjects was compared by Fisher’s two-tailed exact test. A *p* value < 0.05 was considered statistically significant.

### 2.8. Modelling of the Complexes

The EBV-derived epitope (pEBNA3A; RPPIFIRRL) was modelled by means of the mutagenesis tool included in the Pymol software package (The PyMOL Molecular Graphics System. Version 1.7.0.2, Schrödinger, LLC, New York, NY, USA, www.pymol.org), starting from the crystallographic structure of pVIR peptide (RRKWRRWHL) [33]. A lysine residue was added to the pEBNA3A peptide to model the 10-mer peptide (pKEBNA3A; KRPPIFIRRL).

Molecular docking calculations have been performed to verify the peptide binding-groove insertion. The best pose was refined using the Galaxy Refine Complex tool [34] in order to remove unfavorable interactions.

### 2.9. Computational Methods

We performed a molecular dynamics simulation for each of the HLA-B27 subtypes in complex with the corresponding peptide and an additional ligand-free simulation as a control for each of the HLA-B27 subtypes. The simulations have been performed by means of the Gromacs software package (version 5.0.7) [35] using the OPLS-AA force field [36]. All the simulations, lasting ~200 ns each, were performed in a cubic box with the SPC/E water model [37], imposing a distance of 1.2 nm between the protein and the box. The systems were neutralized and simulated at physiological concentration of Na^+^ and Cl^−^ (0.15 M). The temperature was kept constant at 310 K by coupling the system to an external bath [38] with a coupling time constant τT = 0.1 ps. The systems have been coupled to a pressure bath (1 bar) with a coupling time constant τP = 1.0 ps.

The crystallographic structures (PDB 1OGT for B*2705, 1OF2 for B*2709; 1WOV for TIS: B*2705 and 1WOW for TIS:B*2709) were used to start the ligand-free and the TIS-complex simulations. To simulate the EBV-epitope and the complexes formed by the synthetic peptides, a homology modelling procedure has been applied using the corresponding crystal structures (see above).

To evaluate the different subtype-dependent peptide mobility, their entropies (S) have been calculated by means of the Schlitter method [39]. The differences in the peptide entropies between the two HLA-B27 subtypes were computed according to: TΔS = T (S_pep/B*2709_- S_pep/B*2705_).

The principal components (Essential Dynamics, ED) analysis has been performed on the MD trajectories to describe the concerted motions associated to the largest collective atomic fluctuations [40].

## 3. Results

### 3.1. HLA-B27 Allele-Specific Presentation of a Suboptimal EBV Derived Peptide

**Figure 1:**
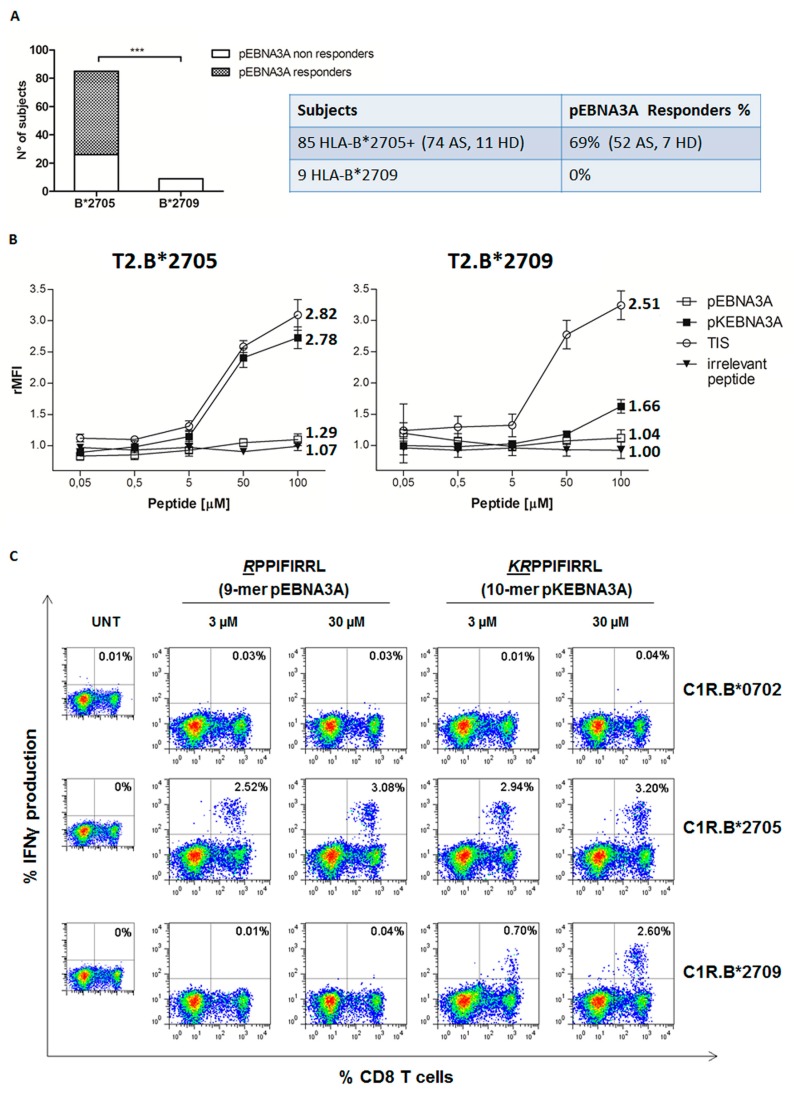
B*2705 but not B*2709-positive carriers mount a response to the 9-mer pEBNA3A epitope. (**A**) CD8+ T cell responses to pEBNA3A detected by IFNγ production analyzed in 85 B*2705 carriers (74 patients with Ankylosing Spondylitis, indicated as AS, and 11 healthy donors, indicated as HD) and in 9 B*2709 healthy donors. 69% of B*2705 carriers but none of B*2709 individuals show reactivity against pEBNA3A antigen (p value < 0.0001 calculated by Fisher’s two-tailed exact test). (**B**) Staining of B*2705 and B*2709 molecules expressed on the cell surface of T2 transfectants performed by using ME1 mAb after 16 h of cell incubation with pEBNA3A, pKEBNA3A, or TIS (RRLPIFSRL) used as positive reference as well as with an irrelevant peptide at the indicated concentrations. Results are expressed as relative mean fluorescence intensity (rMFI) obtained with peptide-treated compared to untreated cells. Values represent the mean ± SEM of three independent experiments. The numbers on the right indicate the fold increase of rMFI obtained with 100 µM compared to 0.5 µM of each peptide. (**C**) Flow cytometry plots reporting IFNγ production by PBMCs from a representative B*2705 positive patient with AS expanded in the presence of pEBNA3A (12 days) and exposed to indicated C1R transfectants pulsed with pEBNA3A or pKEBNA3A (3 or 30 µM) before intracellular staining. Numbers indicate the percentage of IFNγ-secreting CD8+ T cells.

In previous work, we have described in HLA-B*2705 carriers the presence of HLA-B27-restricted CD8+ T lymphocytes specific for an EBV antigen (pEBNA3A; RPPIFIRRL) known as immunodominant in the context of HLA-B7 restriction and suboptimal for HLA-B27. Noteworthy, this occurs in a B27 allele-dependent manner since the same CD8+ T cell reactivity could not be observed in HLA-B*2709 positive healthy donors [31]. To date, we have screened 85 HLA-B*2705 positive individuals (74 patients with AS and 11 healthy controls) and found that 69% of them display pEBNA3A-specific, B*2705-mediated CD8+ T cell responses monitored through the IFNγ production. To this aim, PBMC from these individuals have been cultured in the presence of pEBNA3A (20 μM). After 12 days, they were exposed to pEBNA3A-pulsed C1R.B27 transfectants and the IFNγ production detected in the CD8+ T cell subset (Figure 1A).

As expected, none of the nine B*2709 healthy subjects, analysed in the previous and in the current study, responded to the 9-mer pEBNA3A epitope (Figure 1A).

Next, we asked whether the immune-reactivity against pEBNA3A could be a side-effect of the cross-recognition with the longer natural peptide (pKEBNA3A; KRPPIFIRRL) extended by a Lys at the N-terminus and, therefore, due to the Arg at P2, restoring the canonical B27 consensus motif. At first, we performed cell surface stabilization assays on T2.B*2705 and T2.B*2709 transfectants incubated with increasing concentrations of pKEBNA3A (10-mer), pEBNA3A (9-mer) or TIS used as positive reference. The results in Figure 1B confirmed the inability of pEBNA3A to stabilize either HLA-B*2705 or HLA-B*2709 molecules on B27 transfectants as previously shown in Tedeschi et al. [31]. pKEBNA3A showed instead a dose-response curve nearly overlapping to that of the positive reference TIS peptide on T2.B*2705 cells. When T2.B*2709 transfectants were used, the 10-mer displayed a detectable binding only at the highest dose (Figure 1B). Therefore, pKEBNA3A could be considered as a strong binder for B*2705 and as an intermediate/poor ligand for the B*2709.

PBMC from 15 B*2705 subjects (13 AS patients and two healthy individuals), known to be pEBNA3Aresponders, were then cultured in medium containing pEBNA3A, for 12 days, before re-stimulation with C1R.B*2705, C1R.B*2709 or C1R.B7 cell transfectants pulsed with both pEBNA3A and pKEBNA3A epitopes at 3 or 30 μM. As expected, pEBNA3A triggered IFNγ secretion by CD8+ T cells only when presented by C1R.B*2705 but not by C1R.B*2709 cells (Figure 1C) [31]. Very interestingly, pKEBNA3A was able to activate CD8+ T cell effector functions when displayed in complex with either B*2705 or B*2709 molecules (Figure 1C and Figure S1). This is consistent with the peptide binding data showing a strong binding of pKEBNA3A to B*2705 and a moderate but reproducible stabilization of B*2709 allele. Accordingly, as shown in Figure 1C (right panels), the re-stimulation of PBMC with the 10-mer triggered a slightly higher number of IFNγ producing CD8+ T cells when compared to the shorter pEBNA3A epitope complexed with the B*2705 molecules. This difference, although not statistically significant (*p* > 0.05), is found in 13 out of 15 samples analyzed (Figure S1).

### 3.2. Little Evidence for pKEBNA3A Production in Vivo

The results illustrated above allowed us to speculate that the in vivo antigen processing mechanism could produce pKEBNA3A besides pEBNA3A and that in B27 positive individuals the former antigen is dominant. To better address this point, we asked whether in subjects typed as B*2709 positive, a specific CD8+ T cell response could arise upon stimulation with pKEBNA3A. To this aim, PBMC from 3 B*2709 positive individuals have been analysed for reactivity to the 10-mer pKEBNA3A epitope but in none of them a detectable response was observed (data not shown).

**Figure 2:**
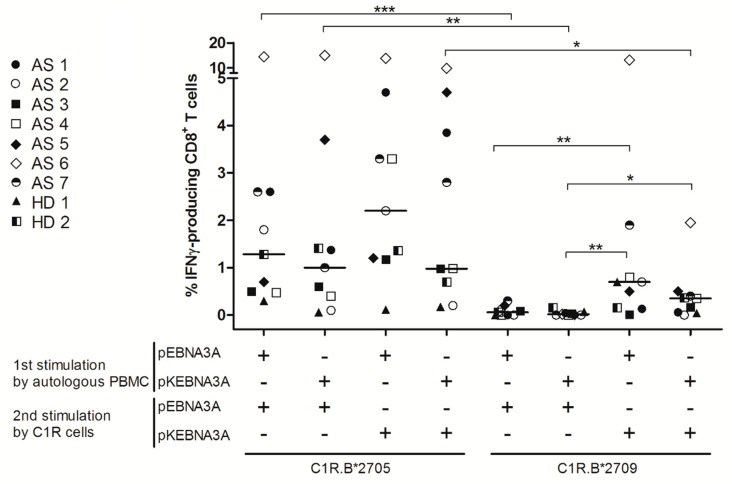
Usage of pEBNA3A in the first stimulation followed by pKEBNA3A as boost leads to the most effective CD8+ T response. IFNγ production by CD8+ T cells was assessed in nine B*2705 positive carriers (seven patients with AS indicated as AS and two healthy subjects indicated as HD). PBMCs were first stimulated with pEBNA3A or pKEBNA3A (12 days) and then re-stimulated with C1R expressing B*2705 or B*2709 molecules, pre-pulsed with the 9-mer or the N-extended version. The percentages of IFNγ-producing CD8+ T cells were compared by Mann Whitney test; *** *p* value < 0.001, ** *p* value < 0.01, * *p* value < 0.05.

A comparative analysis in B*2705 carriers (seven AS patients and two healthy subjects) by a concurrent stimulation of PBMC with pEBNA3A and pKEBNA3A epitopes was then performed. Figure 2 shows a trend of higher magnitude of CD8+ T cell response, monitored as IFNγ producing cells, when the initial antigenic stimulation was done with the shorter peptide. Interestingly, and in agreement with the data shown in Figure 1C, a more effective T cell activation was achieved when the first stimulation was carried out with pEBNA3A and the re-stimulation with pKEBNA3A. Re-stimulation with pKEBNA3A presented by C1R.B*2709 yielded a lower production of IFNγ (Figure 2 and Figure S1). Overall, these results do not support an immunodominance of pKEBNA3A over pEBNA3A.

### 3.3. Peptide Residues Distance from Binding Groove Pockets

**Figure 3:**
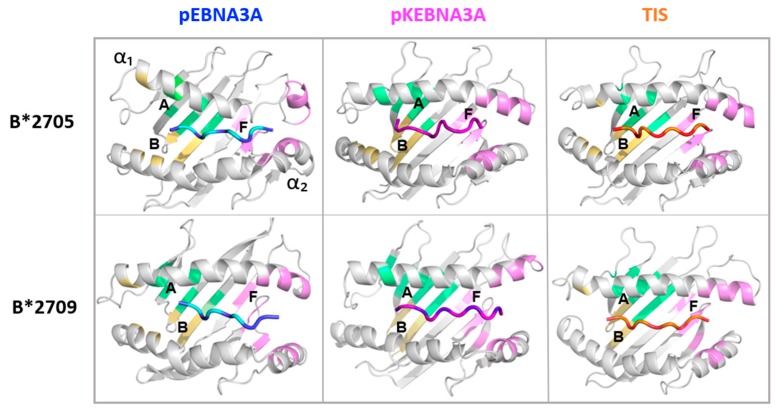
Representative snapshots as obtained by MD simulations of the peptide:HLA-B27 complexes. The upper panels show HLA-B*2705 in complex with pEBNA3A (blue), pKEBNA3A (magenta), TIS (orange) peptides. The bottom panels show the peptide:HLA-B*2709 complexes. A (green), B (yellow) and F (pink) pockets are indicated.

It is known that the HLA peptide is inserted in specific pockets of the binding groove [41]. In particular, the first two amino acid residues are important to anchor the peptide in the A and B pockets of the binding groove. Therefore, to study the conformational behaviour of pEBNA3A and pKEBNA3A in complex with the B*2705 and B*2709 molecules, MD simulations were performed (Figure 3). To understand the peptide positioning in these pockets, the distance of the P1 residue from the central amino acid of the A and B pockets (residues 5 and 45 were chosen, respectively) has been calculated in the case of both peptides in complex with B*2705 and B*2709 molecules. The results shown in Table 1 clearly indicate that, along the MD trajectory, the first residue P1 interacts with the B pocket in both the 9-mer and 10-mer epitopes.

Interestingly, the analysis of the P9/P10 peptide residue interactions shows that the P10 of pKEBNA3A in complex with the B*2709 almost completely filled the F pocket. In all the other systems, the pocket F was mostly unfilled (Table 1).

Moreover, the estimated entropy differences shown in Table 2 revealed higher entropies of the peptides in complex with the B*2705 subtype with respect to the peptides bound to B*2709. This indicates that both peptides, pEBNA3A and pKEBNA3A, are entropically favoured when bound to the HLA-B*2705 with respect to the HLA-B*2709. As a control, we successfully checked that our entropy estimates for the TIS peptide bound to both the B*2709 and the B*2705, were in agreement with a previous computational study [42].

The epitope recognition by TCR might also be influenced by the peptide exposure. However, no relevant differences emerged when the solvent peptide exposure areas of pEBNA3A and pKEBNA3A in complex with the two B27 alleles were compared. As shown in Table 3, the differences between the two subtypes are quite small (within 2 nm^2^), indicating a similar degree of solvent exposure.

### 3.4. Binding Grooves Dynamics

To study the influence of the peptides on the binding groove conformational behaviour, we applied the essential dynamics (ED) analysis to the groove motions.

Starting from the concatenated trajectories of the binding groove residues (residues from one to 175), the ED analysis showed that their motions were described for the 68.5% by the first five eigenvectors.

**Figure 4:**
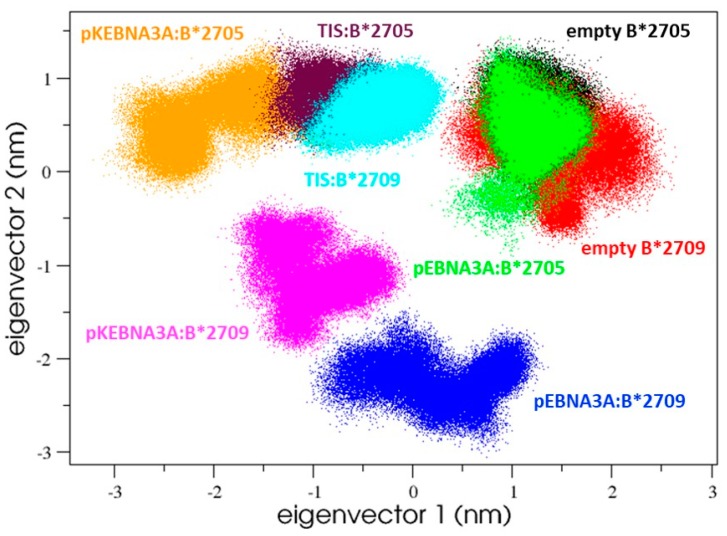
C-alpha binding-groove projections on their common essential subspace, highlighting four main regions featured by: (1) the empty HLA-B*2705/09 and pEBNA3A:B*2705; (2) pKEBNA3A:B*2705 and TIS:B*2705/09; (3) pEBNA3A:HLA-B*2709; (4) pKEBNA3A:HLA-B*2709 complexes. Each point represents a structure sampled by MD simulation.

From the projection of the groove alpha carbons on the first 2 eigenvectors, we obtained the common essential subspace explored by the eight binding grooves, corresponding to the six peptides: HLA-B27 complexes displayed in Figure 4 and the empty B*2705 and B*2709 molecules, used as references.

Interestingly, four main conformational regions sampled by these systems can be identified. One of them characterized the two empty HLA-B27 subtypes as well as the pEBNA3A:HLA-B*2705 complex. Thus, the presence of the 9-mer peptide in the HLA-B*2705 subtype did not seem to modify the groove conformation, being its projection overlapped to the ligand-free one.

In line with our findings, previous computational [42] and experimental [43,44] studies highlighted a subtype-dependent conformational flexibility. In particular, by means of isotope-edited IR spectroscopy [44], observed larger flexibilities and/or an opening of the binding groove for the B*2705 either for peptide-filled or peptide-devoid molecules [43,44]. These observations seem in accordance with the enhanced structural variability observed in the pEBNA3A:HLA-B*2705 complex and reported in Figure 3 (top left panel), where a lack of secondary structure is observed (along the MD trajectory) in a few residues of one helix defining the F pocket.

The second region on such a subspace characterizes the pKEBNA3A:B*2705 and the TIS: B*2705/09 complexes, which are the complexes displaying the highest stability in the binding assay (Figure 4).

The two remaining regions describing the peptide:B*2709 complexes fell in distinct areas of the plane, indicating that both pEBNA3A and pKEBNA3A peptides affected the B*2709 conformational dynamics in different ways (Figure 4).

Additional analysis of the local contacts showed that the P1 residue of the pEBNA3A makes an H-bond with the Glu45 of B*2705 (Table S1). The same behaviour is also observed in the TIS:B27 complexes but not in the pEBNA3A:B*2709 (Table S1).

In summary, the analysis of the binding groove dynamics showed a similar behaviour of TIS: B*2705 and pKEBNA3A:B*2705 complexes indicating similar structural-dynamical effects induced by these peptides on the B*2705. This is consistent with the functional binding data showing that high rMFIs were reached upon binding of both TIS and pKEBNA3A with B*2705 molecules (Figure 1C).

Our data also confirm previous findings suggesting the subtype-dependent conformational dynamics as an intrinsic feature that distinguishes the two HLA-B27 subtypes [42,44]. That is, our computational procedure provides a physically sound explanation of the different binding modes of three peptides bound to either HLA-B27 subtype. However, it should be noted that a generalization of these results as well as the possibility to predict the binding modes by “in-silico” approaches requires several additional MD simulations of different peptides bound to the HLAs, which are beyond the scope of our work and computationally unfeasible.

## 4. Discussion

To our knowledge, this is the first piece of evidence that the HLA-B*2705 is able to unleash a CD8+ T cell response even though presenting a suboptimal viral peptide which possibly occupies the binding groove leaving out the A pocket. This is a relevant observation for an HLA-B allele which predisposes to Spondyloarthritis, particularly to AS, but which is also protective in several viral infections [10,11,12,13]. At first glance, this finding suggests that the actual B*2705 peptidome can indeed be more extended than that detected by high throughput technologies.

The pEBNA3A epitope (RPPIFIRRL), herein investigated, but already described by us [31] as an uncanonical HLA-B*2705 restricted antigen, evokes a CD8+ T cell reactivity in 69% of B*2705 carriers, either patients with AS or healthy controls. This high percentage of pEBNA3A responders substantiates that this peptide can act as an effective antigen in the B*2705 context of presentation although lacking a suitable B27 consensus sequence. As a matter of fact, both mathematical predictive algorithms and experimental binding data have assigned it as a putative non-B27-ligand (Figure 1B) [31]. Therefore, the propensity to load and present suboptimal viral/microbial antigen would be a plausible trait of the HLA-B*2705 by which to broaden the spectrum of triggered immune responses giving a simple immunological explanation for the superior viral immune-surveillance [12,13,14]. It must also be considered that this same feature, through the yield of unstable peptide:HLA complexes, would fuel the tendency of the HLA-B*2705 to produce aberrant forms implicated in the pathogenetic mechanisms of the associated Spondyloarthritis [45].

It is noteworthy that the non-AS-associated HLA-B*2709 allele, which differs from the B*2705 for a single amino acid change in the F pocket [13,25,27], is unable to present this suboptimal epitope [31]. Accordingly, no pEBNA3A-driven CD8+ T cell responses have been found in the nine B*2709 healthy donors screened so far (Figure 1A).

These data prompted us to check whether the CD8+ T cell reactivity towards pEBNA3A in the B*2705 carriers could be a side-effect of the recognition of the longer natural 10-mer (pKEBNA3A). Of note, the N-terminal extension by Lys in pKEBNA3A peptide (KRPPIFIRRL) re-established a typical B27 consensus motif with an Arg in P2. Correspondingly, peptide binding assays on T2.B27 cells pointed out that this N-terminally extended peptide acquired the features of a high and moderate binder for the B*2705 and 09 molecules, respectively (Figure 1B). Nonetheless, functional CD8+ T cell data obtained by stimulating PBMC from B*2705 subjects with the 9-mer and 10-mer in a comparative way and re-stimulating with the same peptides in all four combinations (Figure 2), did not support our hypothesis according to which the latter peptide is immunodominant over the former. In fact, more robust T cell responses, monitored as % of IFNγ-producing CD8+ T cells, have been reached when pEBNA3A was used in the first stimulation. Of note, the best functional outcome was obtained using pEBNA3A in the first followed by pKEBNA3A in the second stimulation. This is most probably due to the higher stability of pKEBNA3A:B27 complexes compared to pEBNA3A:B27 complexes on the C1R transfectants (Figure 2). Overall, these data suggest that the CD8+ T cell repertoire is prevalently pEBNA3A-oriented, most likely because there could be in vivo a sharp prevalence of pEBNA3A:B27 vs. pKEBNA3A:B27 complexes on cell surface. This can be due to the activity of ERAP2, or even ERAP1, which can trim the N-terminal lysine from pKEBNA3A while sparing pEBNA3A from further degradation since the proline at position 2, and the residue immediately upstream of it (arginine), cannot be cut by either aminopeptidase [46] and, therefore, albeit less stable, the pEBNA3A:B*2705 complex results more abundant. The same cannot be seen in the HLA-B*2709 positive subjects, probably due to the lack of flexibility that would be necessary to accommodate the shorter peptide. In principle, the amount of pEBNA3A available to HLA-B27 loading should depend on level of expression [47] and enzymatic activity of ERAP and increases in parallel to it. Notably, AS associates with ERAP1 allelic variants endowed with high enzymatic activity as well as with the presence of ERAP2 [15,16,17]. Hence, we predict that the available quantity of pEBNA3A would be higher in the HLA-B27 positive patients with AS than in B27 healthy subjects. In this regard, it would be informative to look for both pEBNA3A and pKEBNA3A in the B*2705 and B*2709 peptidomes from EBV-B lymphoblastoid cell lines.

Interestingly, we have not detected CD8+ T cell reactivity to either pEBNA3A or pKEBNA3A in the B*2709 healthy donors although the latter peptide can bind and be presented by B*2709 molecules. This has been proved by binding assays (Figure 1B) and by IFNγ production from CD8+ T cells of B*2705 subjects re-stimulated with C1R-B*2709 cells pulsed with pKEBNA3A (Figure 2 and Figure S1). The reason of the lack of such T cell reactivity in B*2709-positive individuals is hard to explain and presumably is a sum of several factors among which the different nature (flexibility/plasticity) of B*2709 versus the B*2705, the availability of these viral peptides influenced by the different ERAP backgrounds (polymorphisms/enzymatic activity) and the mechanisms that shaped the B*2709 vs. the B*2705 restricted T cell repertoire [4,5,14,43,48,49]. Ultimately, this evidence supports the possibility that in vivo pKEBNA3A is absent.

By means of computational analysis, we tried to gain insights into the binding mode of pEBNA3A within the B27 grooves looking for conformational/dynamic peculiarities underlying the different behaviour of the B*2705 and B*2709 pair of alleles in respect to this uncanonical antigen presentation.

As already speculated in the previous study [31] and supported by functional experiments with analogue peptides, MD simulations infer that the pArg1 acts as a principal anchor in both pEBNA3A:HLA-B*2705 and pEBNA3A:HLA-B*2709 complexes making contacts exclusively with the B pocket into the two grooves. As a consequence of this shifting, the peptide is not stabilized into the A pocket which remains empty. Additional MD simulations of different peptide:HLA complexes could be very useful to confirm such a behaviour. However, this peptide conformation has been already disclosed, by the X-ray crystallography, for Tax8 an octameric peptide from human T cell lymphotropic virus-1 when complexed with the HLA-A2 molecule and, more recently, for a truncated 7-mer EBV peptide (LMP2 343-349) in the HLA-A*11:01 antigen-binding cleft [6,50]. Moreover, the peptidome analysis of the B*5101, a HLA class I allele associated with Behçet’s disease, displayed the presence of 10-mers with Pro or Ala at P2 together with their N-terminally truncated forms allowing to speculate about a non-canonical binding mode for these shorter peptides which leaves aside the A pocket [51].

Another important point is that in both pEBNA3A:B*2705 and pEBNA3A:B*2709 complexes, the F pocket is only intermittently filled by the pLeu9 as indicated by large fluctuations observed along the MD trajectories. This implies a remarkable solvent exposition of the C-terminal peptide moiety in both complexes which could favour the interaction with the TCR but it is also suggestive of a rather weak interaction of the peptide into the groove. Considering the T-cell activation data, it is likely that this conformational flexibility at the peptide C-terminus is compensated in the case of the B*2705 through favourable interactions involving the N-terminal moiety of the peptide. Accordingly, pArg1 of pEBNA3A makes a hydrogen-bond with the Glu45 in the B*2705 but not in the B*2709.

Really unexpected is the pKEBNA3A binding mode in the two B27 grooves. Indeed, MD trajectories highlighted a common peptide shift with respect to the shorter pEBNA3A antigen. This means that pLys1, but not pArg2, fits into the B pocket in either B*2705 or B*2709 molecules. Notably, this result is consistent with our recent data showing that also Lys, beside Arg and Gln, could serve as principal B27 anchor residue interacting with the B pocket [21].

The analysis of pKEBNA3A residue interactions showed that the P10, exclusively in the B*2709 binding groove, almost completely fills the F pocket. This further interaction gives a clue to explain why the 10-mer peptide acquires the capability to bind to the B*2709 and be presented to CD8+ T cells inducing their activation. Therefore, the reason of the lack of reactivity in HLA-B*2709 individuals is most probably due to an in vivo shortage of the 10-mer compared to the 9-mer peptide.

Interestingly, the analysis of the groove motions points out that the peptides affect the conformational space accessible to the B27 grooves and, remarkably, a common conformational region is sampled by the three complexes (Figure 4) showing the highest stability induced by the peptide binding (Figure 1B).

Some limitations of this study prevented us to give a definite answer to our hypothesis. First of all, our data neither prove nor disprove the immunodominance of the longer and “canonical” B27-binder peptide (pKEBNA3A) over the shorter and “uncanonical” pEBNA3A antigen. The identification of both or even one of the two peptides in the B*2705 and B*2709 peptidomes from EBV-B lymphoblastoid cell lines would support our hypothesis. Nevertheless, the lack of specific memory T cells in the B*2709 individuals towards the longer peptide which can nonetheless be accommodated in the groove, argues against the *in vivo* availability of this epitope. A second point concerns the intrinsic limitations of the computational methods used to gain insights into the conformations of the two epitopes in complex with both B27 molecules. Unfortunately, a first attempt to make crystals of pEBNA3A and pKEBNA3A in complex with the HLA-B*2705 has been unsuccessful (Dr. Bernhard Loll, personal communication).

In conclusion, this study shows for the first time that the strongest risk factor for AS, i.e., B*2705, is able to elicit anti-viral T cell immune-responses even when the binding groove might be partially occupied by the epitope as inferred from computational analysis. This feature is not shared by the very close B*2709, which is not a risk factor for AS. This evidence hints a deeper definition of the B27 immunopeptidome to gain insights into its pathogenic role in Spondyloarthritis as well as in the immune-surveillance.

## Figures and Tables

**Table 1:** Peptide position in the binding groove.

Peptide: HLA-B27 Complex	P1 Interaction with A Pocket	P1 Interaction with B Pocket	P9 or P10 Interaction with F Pocket
pEBNA3A: B*2705	No	Yes	No §
pEBNA3A: B*2709	No	Yes	No §
pKEBNA3A: B*2705	No §	Yes	No
pKEBNA3A: B*2709	No	Yes	Yes
TIS: B*2705 = TIS: B*2709	Yes	No	Yes
§ high fluctuation			

Peptide interactions in the A and B pockets. P1 and P9/P10 represent the 1st and the last residue, respectively. A distance of 0.4 nm was chosen as interaction cut-off (i.e., “yes” indicates a mean residue distance < 0.4 nm and “no” a mean residue distance > 0.4 nm).

**Table 2:** Peptide entropy differences.

Peptides	TΔS kJ/mol
pEBNA3A: HLA-B*2709-05	−77.6 ± 10.5
pKEBNA3A: HLA-B*2709-05	−47.4 ± 14.7
TIS: HLA-B*2709-05	41.4 ± 1.3

The ΔS is the difference between the entropies of the peptide bound to the B*2709 and that bound to the B*2705.

**Table 3:** Peptide solvent exposure.

Peptides	Solvent Exposure (nm^2^)
pEBNA3A: HLA-B*2705	16.7 ± 0.1
pEBNA3A: HLA-B*2709	16.7 ± 0.1
pKEBNA3A: HLA-B*2705	18.2 ± 0.1
pKEBNA3A: HLA-B*2709	18.9 ± 0.1

## Supplementary Materials

The following are available online at https://www.mdpi.com/2073-4409/8/6/572/s1, Figure S1: B*2709 allele is able to present pKEBNA3A, Table S1. Hydrogen bonds between the peptides and the HLA-B27 binding groove.
