# Supplementary material for: Unusual Placement of an EBV Epitope into the Groove of the Ankylosing Spondylitis-Associated HLA-B27 Allele Allows CD8+ T Cell Activation
Source: Cells. 2019 Jun 11;8(6):572. doi: 10.3390/cells8060572 (PMC6627668; doi:10.3390/cells8060572)
Supplement: Supplementary file 1 [file cells-08-00572-s001.pdf]

Supplementary materials

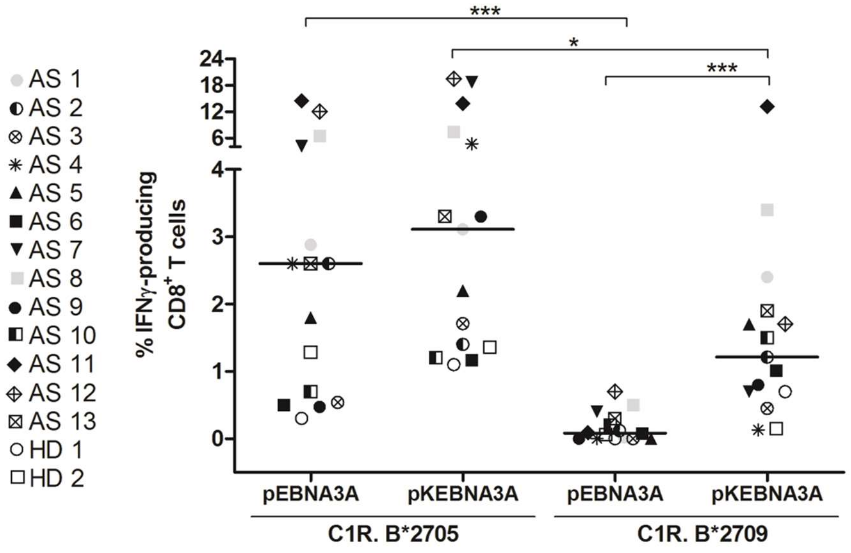

Figure S1. B*2709 allele is able to present pKEBNA3A. PBMC from 15 B*2705-positive subjects (13 patients with AS and 2 HD) were stimulated with pEBNA3A. After 12 days, their activation evaluated through the IFNγ production, was induced by re-stimulation with C1R.B*2705 or C1R.B*2709 transfectants pre-pulsed with pEBNA3A or pKEBNA3A. The percentage of IFNγ-producing CD8+ T cells was compared by Mann Whitney test; ***p value<0.001, *p value<0.05.

Table S1. Hydrogen bonds between the peptides and the HLA-B27 binding groove.

GROOVE	pEBNA3A: HLA-B*2705	pEBNA3A: HLA-B*2709	pKEBNA3A: HLA-B*2705	pKEBNA3A: HLA-B*2709	TIS: HLA-B*2705	TIS: HLA- B*2709
**Thr 24**					P2	P2
**Glu 45**	P1				P2	P2
**Glu 63**	P1	P1	P1, P2		P1, P2	P1, P2
**Arg 62**					P2	P1
**Asp 77**			P9	P9	P8	P9
**Tyr 99**					P3	P3
**Lys 146**					P9	P9
**Trp 147**				P10	P8, P9	P8
**Tyr 159**	P1	P1				P1
**Glu 163**				P2	P1	P1

On the left: amino acid residues in the binding groove involved in the H bonds. The interacting peptide residues are labeled with "P" (i.e. P1 the 1st residue, P9/P10 the last one).
